# Evaluation of mAb 2C5-modified dendrimer-based micelles for the co-delivery of siRNA and chemotherapeutic drug in xenograft mice model

**DOI:** 10.21203/rs.3.rs-3713164/v1

**Published:** 2023-12-11

**Authors:** Satya Siva Kishan Yalamarty, Nina Filipczak, Tanvi Pathrikar, Colin Cotter, Janaína Artem Ataide, Ed Luther, Swarali Paranjape, Vladimir Torchilin

**Affiliations:** 1 Center for Pharmaceutical Biotechnology and Nanomedicine, Northeastern University, Boston, MA 02115, USA; 2 Department of Pharmaceutical Sciences, Northeastern University, Boston MA, 02115, USA; 3 Department of Chemical Engineering, Northeastern University, Boston, MA, 02115, USA; 4 Faculty of Pharmaceutical Sciences, University of Campinas (UNICAMP), Campinas 13083- 871, SP, Brazil

**Keywords:** Co-delivery, siRNA, nanoparticles, *in vivo*, breast cancer, dendrimer, preclinical model

## Abstract

A combination therapy with small interfering RNA (siRNA) and chemotherapeutic drug is proven to be effective in downregulating the cancer resistance proteins, such as P-glycoprotein (P-gp). These proteins are involved in multidrug resistance (MDR) of tumors. MDR lowers the efficacy of chemotherapy and even renders it ineffective. A possible strategy to counteract the resistance is by downregulating the resistance proteins using siRNA. A targeted formulation capable of delivering siRNA and chemotherapeutic drug will not only downregulate P-gp but also increase the concentration of the chemotherapeutic drug at the site of tumor thereby increasing the therapeutic effect and lowering the systemic exposure. In this study, monoclonal antibody 2C5-modified dendrimer-based micelles were used to co-deliver siRNA and doxorubicin (DOX) to the tumor site in both male and female xenograft mice model. The nucleosome-specific 2C5 antibody recognizes the cancer cells via the cell-surface bound nucleosomes. The ability of the 2C5-modified formulation in affecting the metastasis of highly aggressive triple negative breast cancer (MDA-MB-231) was assessed via wound healing assay where the 2C5-modified formulation halved the rate at which the cells were migrating. Further, the therapeutic efficacy of the formulation was assessed by measuring the tumor volume progression where the 2C5-modified nanoparticle group had a similar tumor volume to the free drug group at the end of the study, although a 50% increase in DOX concentrations in blood was observed after the last dose of nanoparticle. Despite a higher DOX concentration and residence time we did not observe any systemic toxicities in the nanoparticle groups. The free drug group on the other hand showed body weight reduction as well as the visible irritation around the injection spot. The treatment group with 2C5-modified micelles has shown to be safe at the current dose of DOX and siRNA.The ability of 2C5 antibody-functionalized nanoparticles in delivering cargo to the tumor site *in vivo* was evaluated for DOX using *ex vivo* imaging and siRNA by western blot study to evaluate the levels of P-gp. Furthermore, the siRNA mediated P-gp downregualtion was studied using western blotting assay. We observed a 29% reduction of P-gp levels in both males and females with respect to the control (BHG). We also conclude that the dose of DOX and siRNA should be further optimized to have a better efficacy in a metastatic tumor model, which will be the subject of our future studies.

## Graphical Abstract

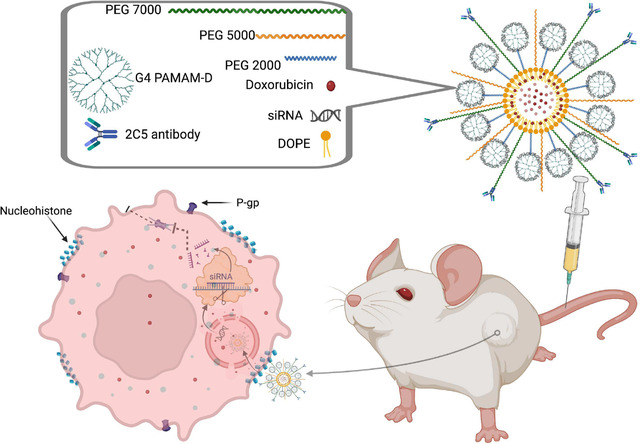

## 1. Introduction

For several decades now, cancer has remained one of the leading causes of death worldwide [1]. Although chemotherapies are available in the market to treat cancer, their effectiveness often remains questionable. The main obstacle to the development of effective cancer treatments is the simultaneous resistance of cancer cells to multiple drugs, known as Multidrug resistance (MDR) [2]. There are several factors responsible for MDR in tumor cells, including altered molecular targets, increased drug metabolism, genetic defects, reduced apoptosis, overexpression of efflux pumps, improved DNA repair pathways, and physiological factors such as cell-to-cell interaction, higher interstitial fluid pressure, low pH environment, regional hypoxia in the tumor, irregular tumor vasculature, and the presence of cancer cells in difficult-to-penetrate areas [3]. Conventional chemotherapies may not be effective against MDR, leading to the application of increased dosages, resulting in increased toxicities, and decreased patient quality of life. Therefore, the counteraction of MDR is required for an effective cancer treatment.

One of the common mechanisms that causes MDR, involves the increased efflux of hydrophobic chemotherapeutic drugs, which is mediated by energy-dependent ATP-binding cassette (ABC) transporters. Several transporters in this class, such as P-gp (P-glycoprotein), MRP1 (multidrug resistance-associated protein-1), BCRP (breast cancer resistance protein), and MXR (mitoxantrone resistance protein), have a broad range of specificities and can promote the efflux of various classes of xenobiotics [4]. P-gp, in particular, is notable since it is responsible for resistance to a wide range of chemotherapeutic agents. P-gp is encoded by the MDR1 gene and is expressed in normal transport epithelium of the liver, kidney, and gastrointestinal tract, where it detoxifies and protects the normal tissues from xenobiotics. However, in tumor cells, overexpression of P-gp results in the reduction in intracellular concentration of chemotherapeutic drugs [2, 5–11].

MDR in tumor cells can be effectively reversed by selectively suppressing the MDR1 gene. Downregulation of P-gp via cancer-specific pathways has been developed to maintain the constitutive expression of P-gp in normal cells [12]. Although small molecule P-gp inhibitors, such as ketoconazole, verapamil, and clarithromycin are available, they can cause side effects and do not downregulate protein expression at the gene level. Recent research has demonstrated that siRNA, an endogenous tool, can effectively downregulate P-gp and lead to MDR reversal [13, 14]. This transcriptional repression strategy is not only highly specific but also aids in preventing P-gp expression during disease progression. However, there are several challenges to delivering siRNA to cancer cells, including poor cellular uptake, rapid clearance, non-ideal biodistribution, and instability [15, 16].

In this study, we evaluated mixed dendrimer-based micelles (MDM) for co-delivery of siRNA and DOX to tumors *in vivo*. Amphiphilic PAMAM-PEG_2k_-DOPE and 1,2-dioleoyl-sn-glycero-3-phosphoethanolamine-conjugated poly(ethylene glycol)5k (PEG_5k_-DOPE) self-assemble into mixed micelles. The PAMAM polymer’s cationic charges bind electrostatically the negatively charged phosphate groups on siRNA, while the lipid core of the micelles can accommodate hydrophobic chemotherapeutic drugs. This allows for the simultaneous delivery of both, the drug and siRNA, to cancer cells and exploits their synergistic effects. Our previous research demonstrated that MDM loaded with DOX and siMDR1 (siRNA specific to MDR1 gene) resulted in a significant downregulation of the membrane-bound P-gp in MDR cancer cells [17]. Typically, nanoparticles are not specific to the tumor site and are cleared quickly from the body. We adopted a targeted delivery approach to enhance the specificity of the MDM [18, 19]. In a previous study, we incorporated the mAb 2C5 into the MDM by conjugating it to a PEG7k-DOPE polymer, and studied the formulation in MDA-MB-231 and SKOV-3TR cell lines [20]. The antibody is specific to cancer cell surface-bound nucleosomes, which results from apoptotically dying neighboring cancer cells [21, 22]. This modification produces the 2C5-modified micelles highly specific to tumor cells and with demonstrate reduced systemic toxicities [23–25]. Additionally, the MDM’s composition can be tailored to suit several functions, enhancing their efficacy further. The MDM’s ease of preparation by simple self-assembly of polymeric components also represents a significant advantage.

In previous studies, we demonstrated that modifying micelles with mAb 2C5 and loading them with DOX and siMDR1 led to improved cellular uptake, cytotoxicity, and enhanced anticancer effects against A2780 ADR ovarian cancer *in vivo* and MDA-MB-231 and SKOV-3TR cell lines in vitro [20, 26]. In this study, we aim to further investigate the safety and efficacy of the platform in MDA-MB-231 (triple negative breast cancer) xenograft murine model. Usually, breast cancer-related studies are performed in females, although rarely breast cancer can occur in males [27], which can certainly justify the use of the male animals in such studies. The hormone independent nature of triple-negative breast cancer allowed us to study the treatment in both males and females thereby allowing to bridge the study gap and also to compare the effect of the therapy on both sexes. The safety of the preparation was evaluated by following the animal weight during the time of dosing along with the subsequent *ex vivo* evaluation of liver weights and levels of liver enzymes in male and female mice. Furthermore, the concentrations of DOX in the blood plasma were evaluated. Additionally, we wanted to evaluate the anti-metastatic potential of the formulation using the wound healing assay (a model of cancer cells proliferation), which is a critical factor of the efficacy of the formulation.

## 2. Materials and Methods

### 2.1 Materials

1,2-dioleoyl-sn-glycero-3-phosphoethanolamine (DOPE) and 1,2-dioleoyl-sn-glycero-3- phosphoethanolamine-N-(methoxy(polyethylene glycol)-5000) (ammonium salt) (PEG_5k_-DOPE) were purchased from Avanti Polar Lipids (AL, USA). PAMAM Dendrimer with an ethylenediamine core, generation 4, 10 % w/w solution in methanol (G(4)-D) and Poly-L-lysine hydrobromide (MW 30,000–70,000) (P2636–25 MG) were purchased from Sigma-Aldrich. Aspartate aminotransferase (AST) Colorimetric Activity Assay Kits was purchased from Cayman Chemical (MI, US). Immuno-compromised NCG mice (strain#572) were obtained from Charles River (MA, US). Doxorubicin, methanol, ethanol, acetonitrile, bovine serum albumin (BSA), Micro BCA assay kit were purchased from Thermo- Fisher Scientific (MA, USA). Triple negative breast cancer MDA-MB-231 cells were purchased from The American Type Culture Collection (Manassas,VA). siRNA targeting MDR-1 (siMDR-1): 5′- GGAAAAGAAACCAACUGUCdTdT-3′ (sense), was purchased from GE Healthcare Dharmacon, (CO, USA). Nuclease-free water was purchased from Qiagen (MD, USA). Dulbecco’s modified Eagle’s media (DMEM), fetal bovine serum (FBS) and Penicillin-streptomycin solution were obtained from CellGro (VA, USA). The Trypan blue solution was obtained from Hyclone (Logan, UT). Antibody 2C5 was from Envigo+. NPC-PEG-2K-NPC (pNP-PEG_2k_-pNP) and NPC-PEG-7K-NPC (pNP-PEG_7k_-pNP) were purchased from Laysan Bio. Triethylamine (TEA) was purchased from Sigma. Cell-culture inserts were purchased from Ibidi. Calf thymus nucleohistone (LS003011) was acquired from Worthington Biochemical Corporation.

### 2.2 Cell culture

The Adriamycin-resistant human breast cancer cell line MDA-MB-231ADR was cultured in DMEM 4.5 g/L glucose. Media was supplemented with 10% FBS and antibiotics (100 IU/mL streptomycin). Both cell lines were cultured at 37 °C with 5% CO2. To maintain the MDR effect the cells were resuspended in media containing 100 nM DOX HCl after each passage [27].

### 2.3 Wound healing assay

MDA-MB-231 cell suspensions grown in T-75 flasks to 85% confluence were harvested and cell suspension of 70 μl was pipetted into the two wells of each insert (15000 cells). Inserts (one per well) were placed in a sterile 24 well dish. Cells were incubated for 24 h at 37°C to allow sedimentation and attachment. The cells were treated with formulation independently at a final concentration of 2.5 μg/mL, The inserts were removed with sterile tweezers and 1 mL medium was added per well to remove unattached cells. One mL of medium was added to the wells before imaging. The morphology of the cells was recorded using a Holomonitor. The wound was imaged holographically over 48 h (at 10-min intervals) to secure quantitative data on gap widths and the percent cell-free areas following the drug treatments. Inserts were introduced inside wells followed by cell seeding, and once a confluent monolayer was produced, the inserts were removed. Thus, the removal of the blockade caused by the insert, resulted in a uniform cell-free zone, into which cells could migrate. The process of migration could be followed by a live-cell imaging technique such as holographic monitoring or using regular microscopy.

### 2.4 Tumor growth inhibition

The experiment on inhibiting tumor growth was conducted on females and males NCG mice, strain 572 aged around 10 weeks with accordance to Northeastern University Animal Care protocol 19–1035R approved by Institutional Animal Care and Use Committee (NU-IACUC). The animals were acquired from Charles River and weighed around 25g to 32g before treatment, females, and males respectively. After an acclimatization period of 7 days, the animals were inoculated with MDA-MB-231 ADR cells in the rear left flank, when the tumors reached approximately 100 mm3, the animals were randomly divided into six groups (n=6). The groups were then administered BHG buffer, 2C5-MDM nanoparticles containing both siRNA and DOX (2C5-MDM-DR), MDM nanoparticles containing both siRNA and DOX(MDM-DR), 2C5-MDM nanoparticles containing only Dox (2C5-MDM-D), Dox solution (DOX.HCl), 2C5-MDM nanoparticles containing only siRNA (2C5-MDM-R) and 2C5-MDM nanoparticles with negative siRNA (2C5-MDM-DR (negative siRNA)). The dose of siRNA was 1.5mg/kg, whereas DOX dose was 0.9mg/kg in all groups. The treatment groups were intravenously injected with 100 μl of the formulation once every three days up to 11 injections. The tumor volume was measured with a Vernier scale and calculated according to the formula: V = 0.5 × W × W×L, where W is the width (smaller dimension of the tumor), and L is the length (the larger dimension). At the end of the experiment, the animals were euthanized, tumors and livers were collected, weighed, and snap-frozen with liquid nitrogen for further analysis.

### 2.5 Blood collection

**Figure 1:**
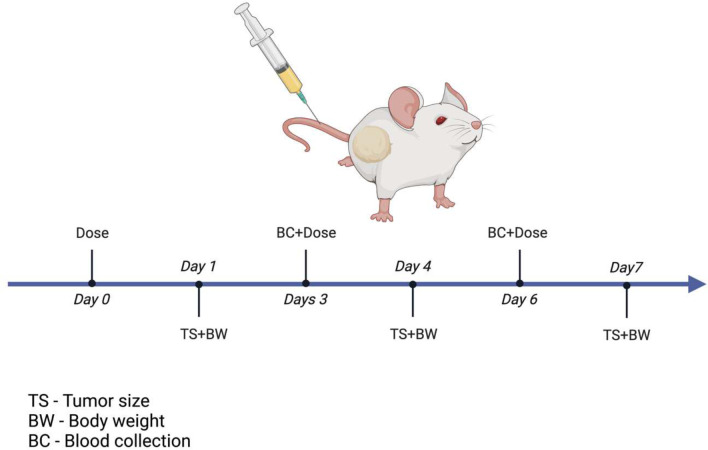
*In vivo* study design. Created by BioRender.com

Blood from the mice was collected by submandibular blood collection method. After administering the first dose blood was collected before administering the subsequent doses. Blood sample was collected from each animal on day 3, day 9, day 15 and day 21 from one group of the animals and from the other group on the day 6, day 12, day 18 and day 24 for a study which was 30 days long. The dosing schedule and blood withdrawal is presented on the Figure 1. Prior to sacrificing blood was also collected via the cardiac puncture method from all mice and centrifuged at 2000 *g* for 30 min at 4 °C to separate the plasma, which was then stored at −80 °C for further analysis of toxicity.

### 2.6 Toxicity

The formulations’ toxicity was determined by monitoring the animals’ weight changes throughout the treatment period. The animals’ weight was measured every other day from the initial injection day until the day of euthanasia. To measure the levels of aspartate aminotransferase (AST) in the plasma, a colorimetric assay kit was utilized, and the manufacturer’s instructions were followed (Cayman, Ann Arbor, MI).

### 2.7 DOX concentration in the blood

DOX concentration in blood was measured by HPLC method. Whole blood samples were mixed with the acetonitrile (40:60) and the fluorescence signal was measured at Ex488 nm/Em560 nm, reversed phase HPLC method on a Hitachi Elute LaChrome HPLC system [28].

### 2.8 Ex Vivo imaging of livers and tumors to evaluate DOX accumulation

The average radiance, which corresponds to the doxorubicin accumulated in the organs was calculated from *ex vivo* fluorescence of tumor and liver tissue using wide-field optical imaging (IVIS Spectrum *In Vivo* Imaging System; Perkin-Elmer, USA). The excitation and emission wavelengths were 480 and 590 nm, respectively. Quantification of fluorescence from tumors and livers is expressed in relative fluorescence units (RFU). The data is presented as the mean ± standard error of the mean.

### 2.9 Western Blotting for siRNA mediated P-gp downregulation

To evaluate P-gp expression, tissue samples underwent lysis in radioimmunoprecipitation assay buffer (RIPA) solution comprising 150 mM NaCl, 25 mM Tris-HCl, 1% Triton X-100, 1 mM EDTA pH = 7.4, 3% sodium dodecyl sulfate (SDS, Thermo Scientific, MA, USA), and 1% sodium deoxycholate, supplemented with 1% protease inhibitor (Thermo Scientific, MA, USA). Protein concentration was quantified using the BCA assay. Equal amounts of protein samples (75 μg) were loaded onto a gel (Novex Wedge-Well 4–20% Tris-glycine gel, Invitrogen, USA) and subsequently transferred to a polyvinylidene fluoride (PVDF) membrane (iBlot, Invitrogen, USA). The PVDF membrane underwent a 12-hour blocking at 4 °C in Tris buffer/Tween 20 (TBST) solution with 3% bovine serum albumin (BSA) (Fraction V) (Thermo Scientific, MA, USA). Subsequently, the membrane was incubated with primary antibodies in TBST solution at room temperature for 2 hours: P-gp (1:2000, Abcam, USA) and β-actin (1:500, ThermoFisher, USA). Following TBST washes, the membrane underwent exposure to secondary antibodies in TBST solution: P-gp (goat anti-rabbit IgG-HRP 1:20 000, Abcam, USA) and β-actin (HRP-conjugated 1:5000, Santa Cruz Biotechnology, Germany) for 1 hour at room temperature. After three TBST washes, the membrane was detected using the SuperSignal West Pico PLUS Chemiluminescence Substrate (Thermo Scientific, USA) for target protein bands following the manufacturer’s instructions.

## 3. Results

### 3.1. Evaluation of tumor cells metastatic potential by wound healing assay

**Figure 2:**
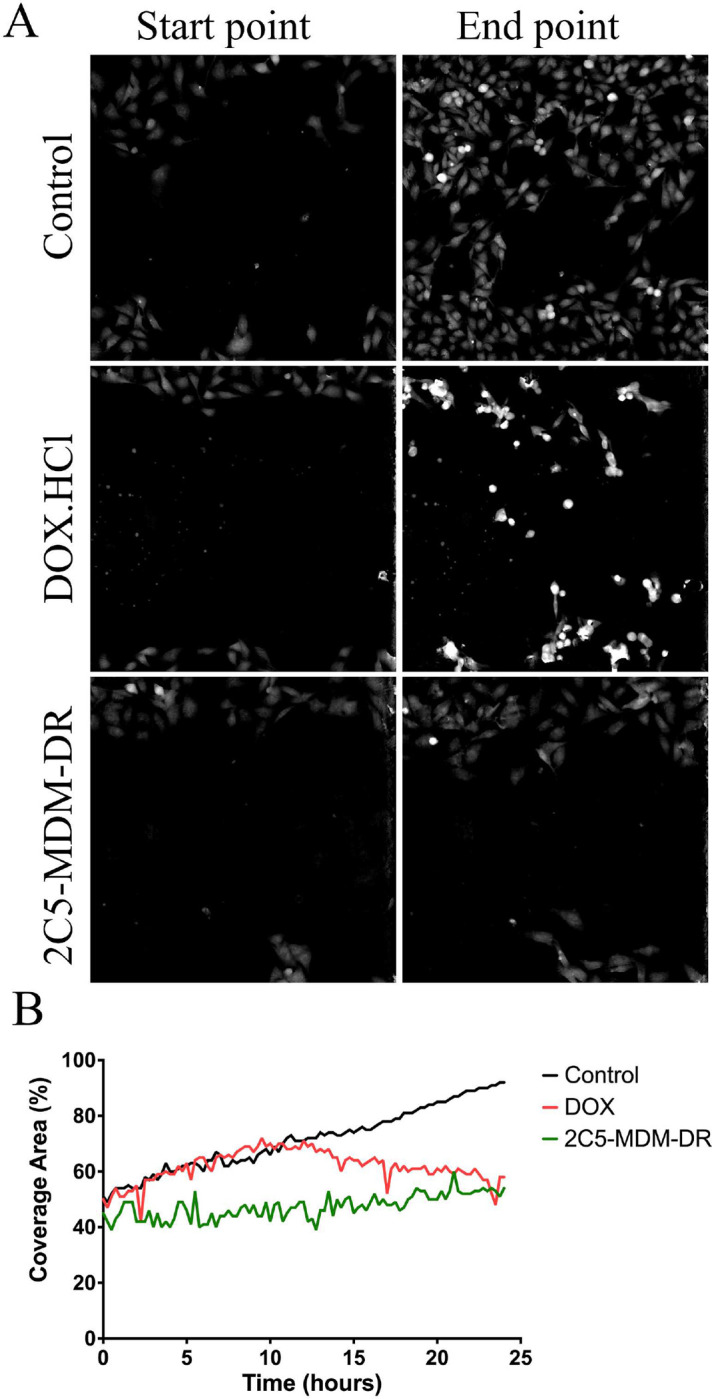
Evaluation of tumor metastatic potential in vitro using wound healing assay, A: Figure shows the start and end points of cell migration in the treatment groups, B: The graph shows the coverage area of the cell with respect to time in hours.

The metastatic potential of MDA-MB-231 cells was evaluated using wound healing assay. Figure 2 shows the migration of the tumor cells *in vitro*. The treatment with 2C5-MDM-DR lowered cell migration, indicative of the treatment potential in metastatic tumors. On the other hand, in the DOX group, a minimal effect on the cell migration was observed at first with the later significant loss of cell migration ability as shown in Figure 2B.

### 3.2 Tumor volume measurement

**Figure 3:**
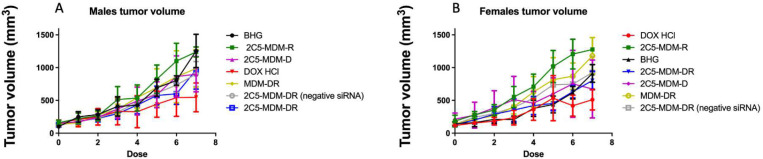
Tumor progression (size) of the xenograft tumors in both male and female mice, A: The data for female mice, B: The data for male mice.

The tumor sizes were measured before every dose administration and the volumes of the tumors were calculated and plotted against time as shown in Figure 3.

Both female and male mice demonstrated similar outcomes in both, DOX, and 2C5-MDM-DR treatment groups. Similar effect was achieved with similar DOX dose. Worth mentioning that our previous study revealed a synergistic effect for the 2C5-modified formulation loaded with DOX and siRNA [20].

### 3.3 Toxicity of DOX according to the body weight measurement

**Figure 4:**
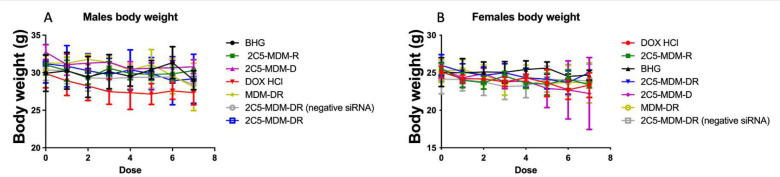
Body weight change of both male and female mice, A: The data for female mice, B: The data for male mice.

**Figure 5:**
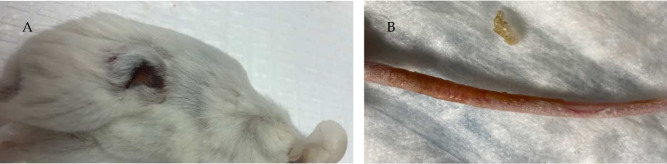
Dermatological toxicities observed in free DOX treated group, A: Open wounds on the mice in DOX group, B: Skin peeling observed on the tail region of the mice after repeated dosing.

To evaluate the toxicity of the treatment, the weight of each animal was measured prior to every administration. Figure 4 shows the data of the body weight changes in both male and female mice. We notice there is a decrease in the weight of the animals particularly in the DOX group. This body weight reduction was more pronounced in the male mice versus the female mice. The DOX group also showed significant toxicities such as skin peeling on the tail and open wounds indicating local toxicities (Fig.5). At the same time, 2C5-MDM-DR demonstrated minimal toxicities in experimental animals, indicating that a similar dose of DOX is safe when administered via the 2C5-modified nanopreparation.

### 3.4 Ex vivo liver and tumor weights

**Figure 6:**
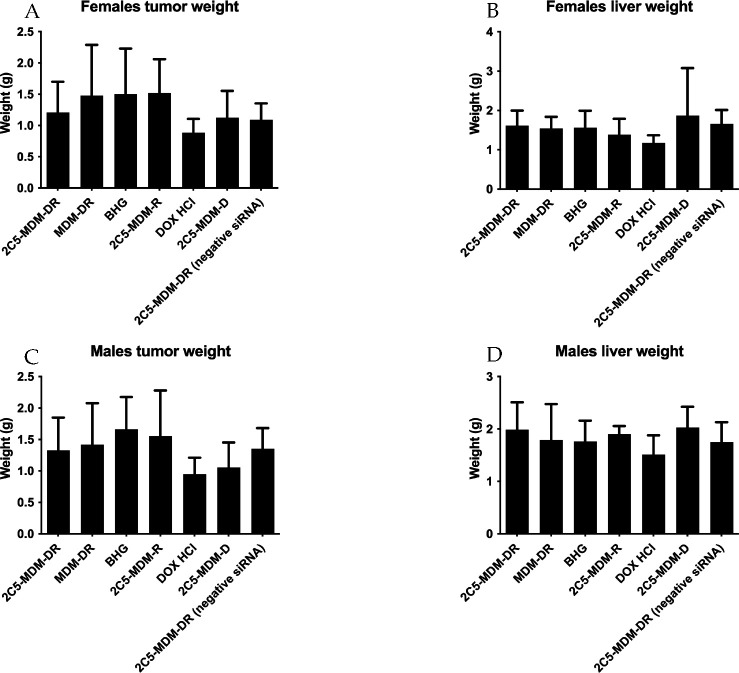
Weights of harvested tumors and livers in female and male mice. The tumors weights show the effectiveness of the treatment and the weights of the liver show the safety of the treatment, A: Tumor weights in female mice, B: Liver weights in female mice, C: Tumor weights in male mice, D: Liver weights in male mice.

To further evaluate the safety and efficacy of the treatments, the weights of harvested tumors and livers were measured *ex vivo* for both male and female mice. The DOX group demonstrates drug toxic effect, since the weight of the livers are much lower than in other treatment groups in both male and female mice (Figure 6). Although the weight of male livers is higher in general the DOX group demonstrated the lowest weight among all the treatment groups. Although, the weights of the tumors are the lowest for DOX group, still this group shows significant toxicities. On the other hand, 2C5-MDM-DR group has healthy livers similar in the control (BHG) group. The weight of tumors in the 2C5-MDM-DR group are lower than in the non-targeted MDM-DR group in both male and female mice.

### 3.5 AST levels in plasma

**Figure 7:**
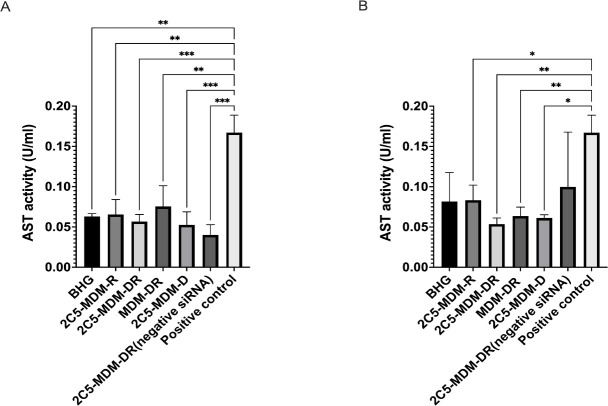
Liver enzyme Aspartate Aminotransferase Activity (AST) activity indicates the safety of the treatment and liver toxicity caused by the treatment, A: AST activity in male mice, B: AST activity in female mice. Results indicate ± SD (n = 3), and significance was calculated with one-way ANOVA comparisons. **** p ≤ 0.0001, *** p ≤ 0.001, ** p ≤ 0.01, * p ≤ 0.05.

To further evaluate if the treatment affects the liver function and demonstrates liver toxicities, Aspartate Aminotransferase (AST) levels were estimated. There were no signs of significant toxicity in both, females and males as evidenced by the AST levels. Whereas the positive control has the highest AST levels in comparison to other groups, still the changes are insignificant. Interestingly, as shown in the Figure 7, the AST levels in females are somewhat higher than in males.

### 3.6 Drug accumulation

#### 3.6.1 DOX concentration in the blood

**Figure 8:**
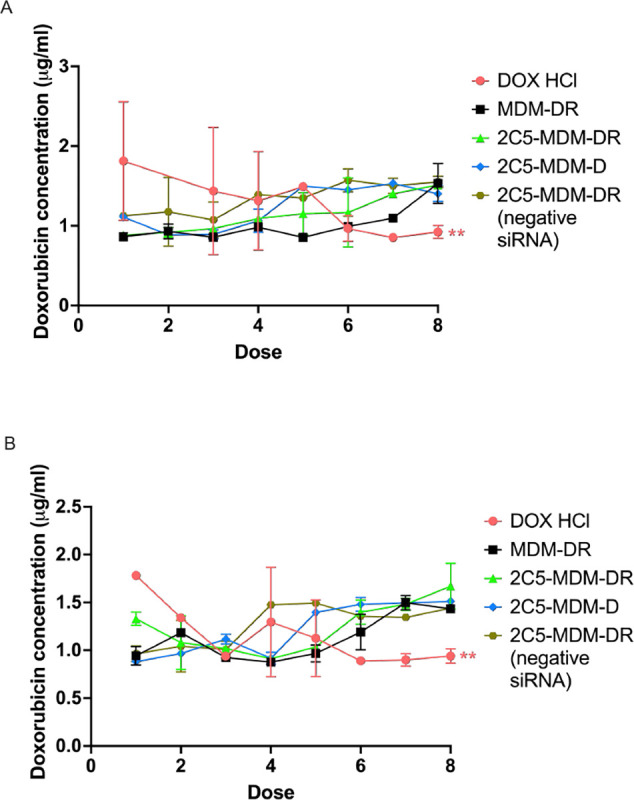
Plasma concentrations of DOX prior to administration, A: Plasma concentrations of DOX with time in male mice, B: Plasma concentration of DOX with respect to time in female mice. Results indicate ± SD (n = 3), and significance was calculated with one-way ANOVA comparisons. ** p ≤ 0.05.

The plasma concentrations of DOX were quantified using HPLC to evaluate the trough concentration of the drug. The 2C5-MDM-DR group in comparison with DOX group has shown higher long-term drug accumulation. In DOX group, the drug is cleared faster than the nanoparticle group. Figure 8 shows the data for both, males and females.

#### 3.6.2 Evaluation of DOX accumulation by ex vivo imaging of harvested livers and tumors

**Figure 9:**
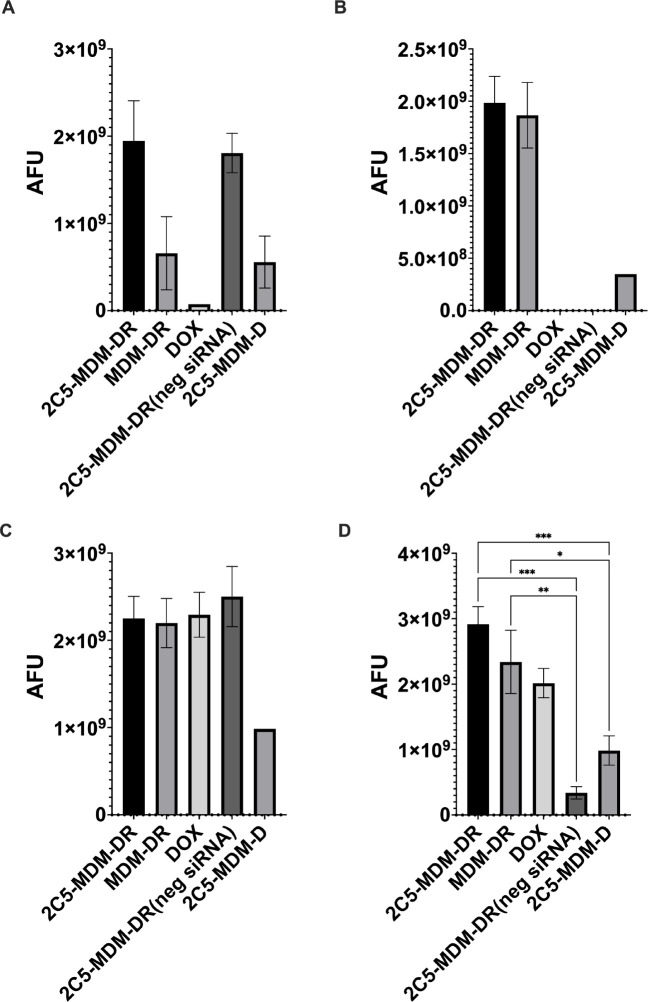
DOX accumulation from the *ex vivo* imaging data of the tumors and livers of female and male animals, A: Female tumors, B: Female livers, C: Male tumors, D: Male livers. Results indicate ± SD (n = 3), and significance was calculated with two-way ANOVA comparisons. **** p ≤ 0.0001, *** p ≤ 0.001, ** p ≤ 0.01, * p ≤ 0.05.

To study the DOX accumulation, *ex vivo* imaging of the harvested tumors and livers of both males and females were performed. Figure 8 shows the accumulation of DOX fluorescence in the different treatment groups. The overall drug accumulation in the females is lower than in the males. The tumors in females had high drug accumulation for the 2C5-MDM-DR group (drug load consists of siRNA and DOX). The DOX group itself provides very low accumulation of DOX in the tumors of females compared to the males. 2C5-MDM-DR application also resulted in higher drug accumulation in the female livers. In part, those differences can be attributed to the autofluorescence from the metastatic tumor tissue in the liver which is more pronounced in females. No DOX accumulation was observed in the female livers for the DOX and 2C5-MDM-DR (neg siRNA) groups. High accumulation of DOX was observed in male tumors in every treatment group except 2C5-MDM-D. Both tumors and livers have shown high accumulation for 2C5-MDM-DR. The livers have higher accumulation of the 2C5-MDM-DR which can be attributed to the metastatic tumor in the liver tissue as shown in Figure 9.

### 3.7 siRNA mediated P-gp downregulation in tumors

Western blot was performed on the harvested tumors to study the downregualtion of P-gp in the tumors. Figure 11A shows the P-gp levels in female tumors. The BHG groups serves as the control and we can observe that there is a considerable downregualtion of P-gp in the treatment groups versus the control. The highest downregulation was observed in the siRNA loaded nanoparticles (2C5-MDM-R). Both non-targeted formulation and the negative siRNA groups do not have a considerable downregulation which was expected. The 2C5-modified nanoparticles loaded with siRNA and DOX (2C5-MDM-DR) have a similar level of P-gp downregulation as 2C5-MDM-R. Figure 11B shows the P-gp levels in male tumors . It is interesting to note that the overall P-gp levels in males are lower than in females. We observed a considerable downregualtion of P-gp in the 2C5-MDM-DR treatment group in comparison to the control (BHG group).

### 3.8 Gender differences

**Figure 10:**
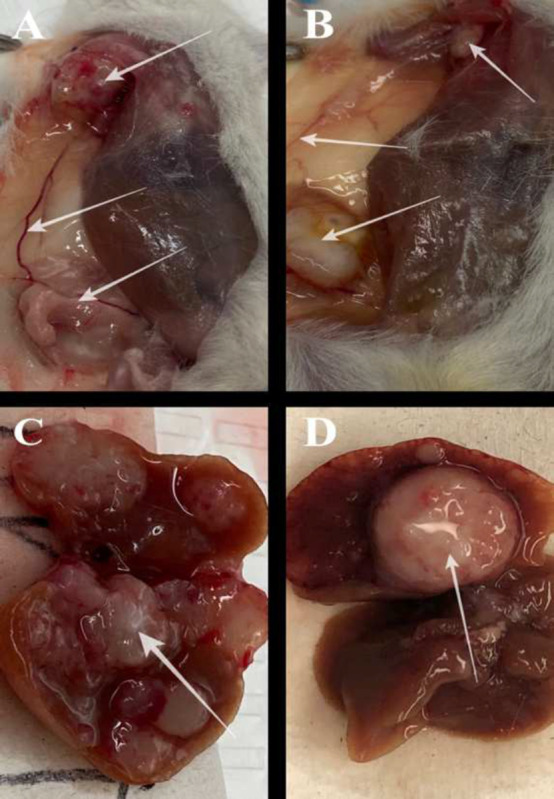
Tumor metastasis to the liver and other regions in the body, A and B: multiple tumors in the neck region that were metastasized from the primary tumor and a blood vessel connecting them, C and D: Livers with metastatic tumor.

Gender differences is an important consideration in the study. Primarily, because the toxicities caused by chemotherapeutic agents, such as DOX may vary with gender. Figure 3 suggests that males in DOX group demonstrated a noticeable weight loss in comparison to the other treatment groups. However, in both males and females other signs of toxicity, such as open wounds and peeling of the skin and poor luster of the fur of the animals in the DOX group were observed. Another observation was the AST data, where the females had a higher baseline level of AST and higher elevation of the liver enzyme in comparison to their male counterparts as shown in Figure 6. Both males and females have shown greater clearance of DOX and higher accumulation and residence time for the 2C5-modified micelles as shown in Figure 7. Little to no fluorescence of DOX in the tumors and livers of females is indicative of higher renal clearance of DOX in females in comparison to males, which demonstrated high drug accumulation. In both male and female subjects, the treatment with 2C5-MDM-DR led to a higher degree of tumor metastasis to the liver as evidenced by increased fluorescence of DOX in both tumors and livers (Figure 9 and 10). Lastly, the P-gp levels in both males and female treatment groups were analyzed using western blot experiment and it is interesting to note that the baseline P-gp levels of tumors in male mice were considerably lower in comparison to the tumors in female mice.

## 4. Discussion

The metastatic potential of MDA-MB-231 tumor cells was assessed through a wound healing experiment. The wound healing assay is a rapid and straightforward *in vitro* technique to examine cell migration [29]. Figure 2A illustrates the images of control, DOX, and 2C5-MDM-DR groups based on the refractive index, and the Figure 2B shows the representation of the area covered by the migrating cells into the gap. The cells were treated with 50nM concentration of siRNA and 3.4nM of DOX. At low concentration of DOX, cells in the free drug group show shrinkage indicative of cell death whereas in the 2C5-MDM-DR group the cells seem to survive and show an effect in slowing the migration from the onset unlike in the free drug group. A difference in cell migration pattern was observed between the DOX treatment and the 2C5-modified nanoparticle treatment. The cells in the DOX group are denser which is indicative of cell death. The nanoparticle treatment group demonstrated slow and steady cell growth and migration compared to the control group. On the other hand, the cells in the DOX treatment group appeared to grow and migrate rapidly at first like in control group, but then slowed down. The rapid initial migration is due to insufficient drug accumulation within the cells due to overexpression of drug efflux pumps which are one of the reasons for multidrug resistance. Since the experiment is done in a closed system, the drug effect takes place when enough time is given which is further demonstrated by the slowing down of cell migration. The slow and lowered gap coverage in the 2C5-MDM-DR group relative to control group indicates the potential of 2C5-modified nanoparticle to lower cell migration, i.e. to show the anti-metastatic effect.

In our previous studies, we were able to establish the stability and safety of the 2C5-MDM-DR formulation, making it suitable for *in vivo* testing [20]. This paper we continue to evaluate the therapeutic potential of the 2C5-modified nanopreparation in a xenograft animal model (MDA-MB-231). In our previous study involving a xenograft ovarian tumor model, the targeted formulation was able to induce a significantly higher tumor growth inhibition compared to DOX and non-targeted formulation[30]. Here, in the current xenograft mice model involving MDA-MB-231, we were not able to observe a significant difference in therapeutic potential between the DOX and 2C5-MDM-DR group in both the genders (Figure 3). Although DOX-treated animals showed the lowest weight of harvested tumors (Figure 5) in both males and females; however, this was accompanied with more pronounced body weight loss (Figure 4), when compared to all other treatments, especially in males. In both sexes, significant cutaneous toxicities were observed in the DOX-treated group. Earlier studies have shown that targeting liposomal DOX formulation with 2C5 antibody had a positive effect in reducing the frequency of auricular erythema in tumor-bearing mice by three to four-folds, due to lower accumulation in the skin [31]. Similarly, in our study we have noticed the 2C5-modified nanopreparation to be safer than the DOX. Earlier studies have shown severe dermatological toxicities even with liposomal DOX treatment [32] however, in our 2C5 modified nanoparticle preparation we have observed none of these dermatological toxicities. Nonetheless, we observed such dermatological toxicities in the free drug (DOX) group alone. This further illustrates the safety of the 2C5-modified nanoparticle preparation under study.

Concentrations of DOX in the whole blood of both, males, and females, were quantified using HPLC. Figure 8A and 8B clearly demonstrate that in free DOX group the drug was clearing more rapidly than in the case of the DOX-loaded micelles. We have observed a cumulative effect of DOX in the 2C5-MDM-DR group, leading to an increase in the drug’s residence time. After the initial dose, DOX exhibited a higher concentration in the bloodstream, which gradually decreased with time and subsequent doses. The steady-state concentration of DOX was never achieved in the free DOX group. In both, males and females, we observed a significant DOX clearance with respect to the DOX levels in the nanoparticle’s groups. One significant finding was that in males, consecutive doses of 2C5-MDM-DR led to a steady accumulation of DOX. However, in females, DOX from the formulation was initially cleared out, with an increase in accumulation was observed only by dose 8. The nanoparticle formulation and free DOX were injected intravenously (I.V.) via the tail vein injection. Most of the DOX injected IV is cleared within a day [33]. The data demonstrated the decreased clearance of DOX in the DOX-loaded nanoparticle group and therefor increased residence time of the drug.

To evaluate the DOX accumulation in different treatment groups, *ex vivo* imaging of tumors and livers was performed in both male and female mice after the organs were harvested. Figures 9A and 9B show DOX accumulation in female tumors and livers, respectively. In both female tumors and livers, there was a significant accumulation of DOX in the 2C5-MDM-DR group compared to the other treatment groups. Similarly, Figures 9C and 9D show the DOX accumulation in male tumors and livers, respectively. A higher DOX accumulation was observed in both male tumors and livers in the 2C5-MDM-DR group. 2C5-MDM-DR resulted in a significant DOX accumulation in male liver in comparison to other treatment groups, highlighting the specificity of 2C5 antibody to the tumors. Interestingly, males had a higher DOX accumulation than females across all treatment groups. Female tumors and livers showed lower DOX accumulation than their male counterparts, possibly due to higher DOX renal clearance. The MDA-MB-231 cells, a highly aggressive and metastatic triple-negative breast cancer line, demonstrate their metastatic nature (Figure 10). The cancer cells have migrated from the primary tumor to different tissues and parts of the body, including the liver, causing extensive metastases. Additionally, the increased accumulation of 2C5-MDM-DR in the livers of both males and females can be attributed to the high specificity of the 2C5 antibody to the tumor site. [34, 35].

Figures 10A and 10B demonstrate the tumor’s metastasis from the primary site to the lymph node where the secondary tumor was noticed. Metastatic tumors were observed in several animals, both male and female. Figures 10C and 10D display representative images of metastatic tumors in the liver. The MDA-MB-231 tumor used in the xenograft model is well-known to metastasize to various organs, including the liver, lymph nodes, lungs, bones, and brain [36]. Although the nanoparticle formulation inhibited cell migration *in vitro*, a similar effect was not observed *in vivo* at the current dose. The dose administered in this study might be too low for the aggressive metastatic triple-negative breast cancer. In our previous *in vivo* evaluation, a significant reduction in tumor volume was observed in a xenograft ovarian tumor model with even lower dose than currently used (0.9 mg/kg dose of DOX and a 1.2 mg/kg dose of siMDR1). However, the lack of significance in tumor volume reduction in this current study can be attributed to the aggressive nature of the triple-negative breast cancer model.

**Figure 11:**
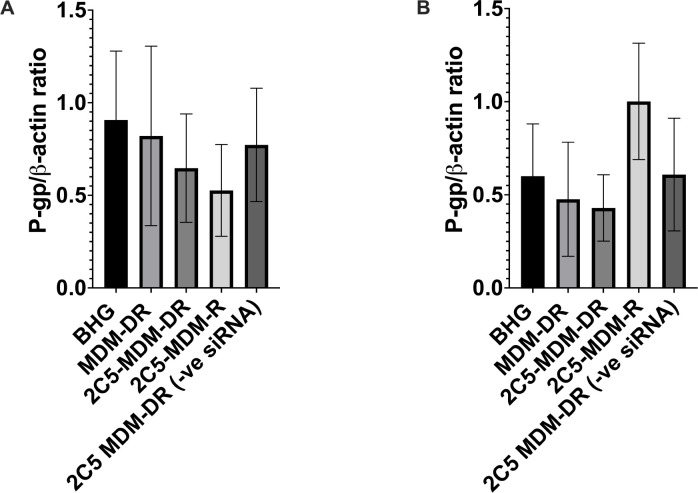
P-gp levels in harvested tumors measured using western blot. A. P-gp levels female tumors, B. P-gp levels in male tumors. The above data shows the densitometry analysis. Results indicate ± SD (n=3).

To evaluate the siRNA mediated P-gp downregulation, we performed a western blot study in the harvested tumors of both males and females. It is interesting to note that the baseline levels of males are lower in comparison to the females as shown in Figure 11A and 11B. More importantly we observed a considerable downregulation of P-gp levels in the treatment groups in comparison to the control. The 2C5-MDM-DR and 2C5-MDM-R treatment groups in the female mice have shown considerable downregulation of P-gp with respect to the control while 2C5-MDM-DR group in the male mice have shown considerable downregulation of P-gp levels. The negative siRNA group in males have shown similar P-gp levels to the BHG group which shows the ineffectiveness of the scrambled siRNA in targeting P-gp. Contrary to females the 2C5-MDM-R treatment group in males is rather ineffective. Although a statistical significance was not observed in the study, we could clearly notice a trend in the results showing siRNA mediated P-gp downregulation.

Previous studies have highlighted gender differences in chemotherapy, with research indicating that sex-specific differences in metabolic enzymes and transporters in the liver and kidneys can result in unique pharmacokinetics for commonly used anticancer drugs [37]. In the present study, DOX-treated animals in both genders displayed lower liver weight (Figure 5) compared to other treatment groups. This can be an indicator of acute drug toxicity in the DOX-treated animals. Even though no significant elevations in AST levels were noticed, female animals exhibited higher baseline AST levels than males across all treatment groups (Figure 6). These finding align with previous research that suggests these drugs often have an extended half-life in women, which has been linked to both improved survival rates and increased toxicity [37].

## 5. Conclusions

In our current study, we further evaluated the mAb 2C5-modified mixed dendrimer micelles as drug/siRNA carriers in the MDA-MB-231 (triple negative breast cancer) xenograft mice model. The wound healing assay results suggests that the migration of tumor cells treated with 2C5-MDM-DR had slowed down in comparison to the control, indicating that the formulation lowers the metastasis. However, the presence of extensive metastasis of the cancer in the liver and lymph node was still observed *in vivo*, probably due to the insufficient drug dose required for this difficult to treat cancer The *ex vivo* imaging data revealed that the formulation is specific to both primary and metastatic tumors. The concentration of DOX in the blood indicates that the 2C5-MDM-DR formulation increases the drug’s residence time. The tumor volume progression, body weight changes and weight of harvested organs indicate that current dose of 2C5-MDM-DR formulation demonstrated similar efficacy as the same dose of free DOX, but without any dermatological toxicities noticed in the free drug (DOX) group. Moreover, there were no considerable increases in AST levels, indicating that the formulation is safe with no liver toxicities. The DOX concentration in the blood suggests that the formulation increases the residence time of DOX in the body. The current data suggests that the treatment at the current DOX dose is safe, however, further optimization of the dose to have a higher efficacy in a metastatic tumor model is needed. In future, we would like to run a dose screening to optimize the dose of the formulation for the metastatic model. In conclusion, although no considerable improvement in efficacy of the 2C5-modified formulation was observed in comparison to the free drug group, the 2C5-modified nanoparticles are found to be considerably less toxic.

## Data Availability

The data that support the findings of this study are available from the corresponding author, V.P.T, upon reasonable request.
